# Familial non-obese idiopathic intracranial hypertension

**DOI:** 10.1016/j.ajoc.2022.101619

**Published:** 2022-06-16

**Authors:** Raed Behbehani, Abdullah Ali, Ashref J. Al-Mousa, Sarah N. Albuloushi

**Affiliations:** Al-Bahar Ophthalmology Center, Ibn Sina Hospital, P.O Box 1180, Kuwait

**Keywords:** Idiopathic intracranial hypertension, Papilledema, Familial idiopathic intracranial hypertension

## Abstract

**Purpose:**

To report a series of cases of non-obese familial idiopathic intracranial hypertension.

**Observation:**

One father and three offsprings (two brothers and one sister) with idiopathic intracranial hypertension and different phenotypic presentations.

**Conclusion and Importance:**

Familial idiopathic intracranial hypertension may underrecognized and may not be associated with obesity. Symptomatic family members may need to be screened for IIH in some cases.

## 1 Introduction

Idiopathic intracranial hypertension (IIH), also known as pseudotumor cerebri, is characterized by papilledema and signs of increased intracranial pressure such as diffuse headache, transient visual obscurations, pulsatile tinnitus, nausea, and vomiting.^1^ The disease predominantly affects young women in their reproductive age and is strongly associated with obesity or recent weight gain.^1^ The diagnosis is made by fulfilling the modified Dandy's criteria.^2^ Familial IIH is rare with only a few case reports and series in the literature with most of the cases reported were associated with obesity in family members with variable patterns of inheritance.^3^ We report three cases of non-obese siblings of two brothers and a sister and their father diagnosed with IIH with variable phenotypic presentation and review the pertinent literature and the possible etiology. This report was published with the permission and informed consent of the patients.

### 1.1 Case 1

The father of cases 2–4 was diagnosed with IIH 22 years ago as he presented with transient visual obscurations in his left eye and headache. He was not reported to be obese nor had recent weight gain before the onset of symptoms with no optic disc swelling in both eyes. His MRI was normal and his lumbar puncture showed an opening pressure of 44 cmH2O with normal CSF composition. He was treated with acetazolamide1 gram/day but then subsequently underwent ventriculoperitoneal shunt placement in 2001 because of deteriorating vision. His final visual acuity was 20/200 OD and 20/HM OS, and he had a left RAPD. Fundus examination showed right grade 2 Frisen papilledema, and left optic disc pallor.

### 1.2 Case 2

**Fig. 1:**
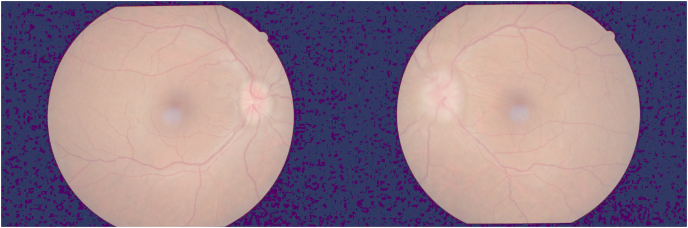
Fundus photograph showing bilateral papilledema more severe in the left eye.

**Fig. 2:**
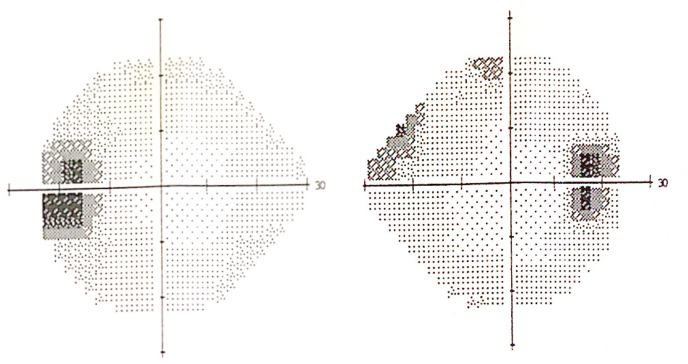
Humphrey visual field testing (24–2) shows enlarged blind spots in both eyes.

**Fig. 3:**
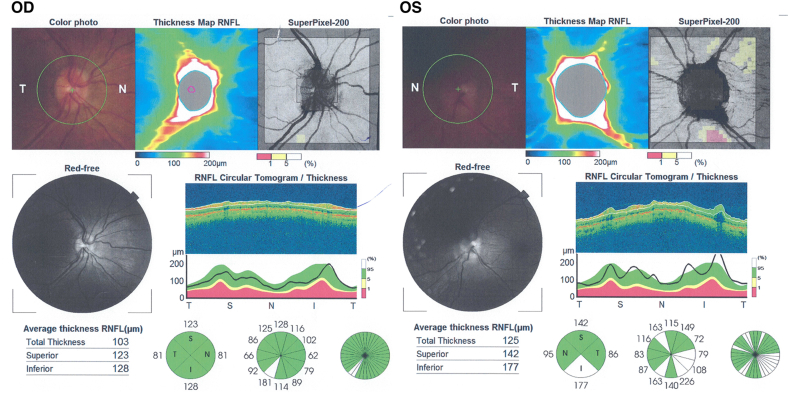
Spectral domain optical coherence tomography (Cirrus 5000) shows normal retinal nerve fiber layer thickness in the right and mild thickening of retinal nerve fiber layer in the left eye.

A 38-year old man presented with a two-month history of transient visual obscurations in the left eye associated with generalized headache and pulsatile tinnitus. His weight was 73 kg and his height was 177 cm (Body-mass index 23.5). His past medical history was non-contributory and he was not using any medications. The patient's visual acuity was 20/20 OU and color vision testing using the pseudoisochromatic plates was full in both eyes. His pupillary reactions were brisk with no afferent pupillary defect. Fundus examination showed bilateral papilledema more severe in the left than the right eye and optic disc drusen excluded (Fig. 1). Visual field testing showed enlarged blind spots in both eyes with nasal step in left eye(Fig. 2). MRI/MRV of the brain was normal and lumbar puncture showed an opening CSF pressure of 36 cmH2O and normal CSF analysis. He was started on oral acetazolamide 1.5 g/day with minimal improvement in papilledema over the course of his follow up and he continued to experience frequent transient visual obscurations. Three months later his papilledema persisted with no improvement and he became intolerant to higher doses of acetazolamide, therefore optic nerve sheath fenestration was performed for his left eye followed for the right eye. At his last follow-up, the papilledema in the right eye had resolved, while there was mild disc swelling in the left eye (Fig. 3).

### 1.3 Case 3

**Fig. 4:**
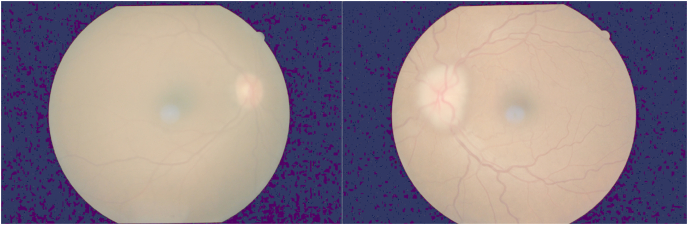
Fundus photograph showing bilateral papilledema more severe in the left eye.

**Fig. 5:**
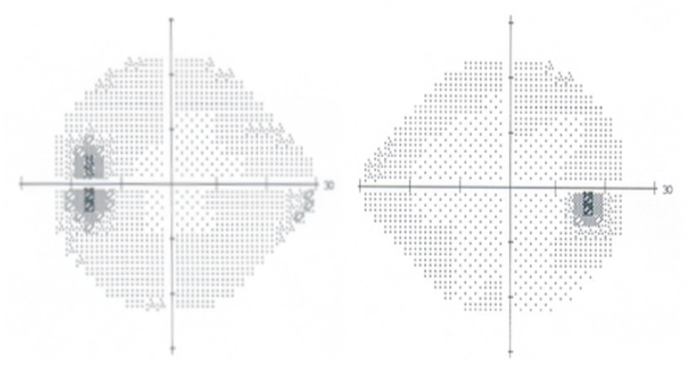
Humphrey visual field testing (24–2) shows enlarged blind spot in the left eye and normal field in the right eye.

A 41-year old woman, sister of cases 2 and 4, presented with recent progressive transient visual obscurations in the left eye with headache with pulsatile tinnitus. Her weight was 54 kg and her height was 162 cm with (Body mass index of 20.6). The patient denied any use of any medication including tetracycline or any Vitamin-A derivatives. Her visual acuity was 20/20 OU and color vision testing was full in the right eye and she recognized 14 of 15 plates in the left eye. Pupillary reactions were normal and fundus examination showed optic disc swelling in the left eye only and normal optic nerve appearance in the right eye (Fig. 4). Visual field testing showed an enlarged blind spot in the left and was normal in the right eye (Fig. 5). MRI/MRV of the brain was normal. Lumbar puncture was done in lateral decubitus position the and the CSF opening pressure was 26 cmH2O with normal composition.

### 1.4 Case 4

**Fig. 6:**
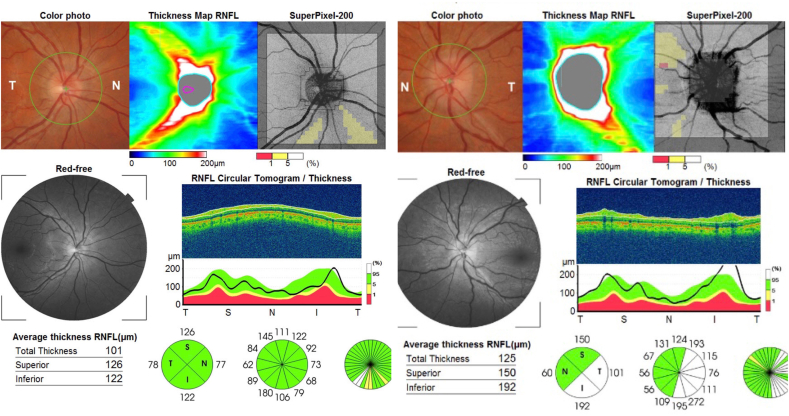
Spectral-domain optical coherence tomography (Cirrus 5000) shows increased retinal nerve fiber layer thickness in the left eye and is normal in the right eye.

**Fig. 7:**
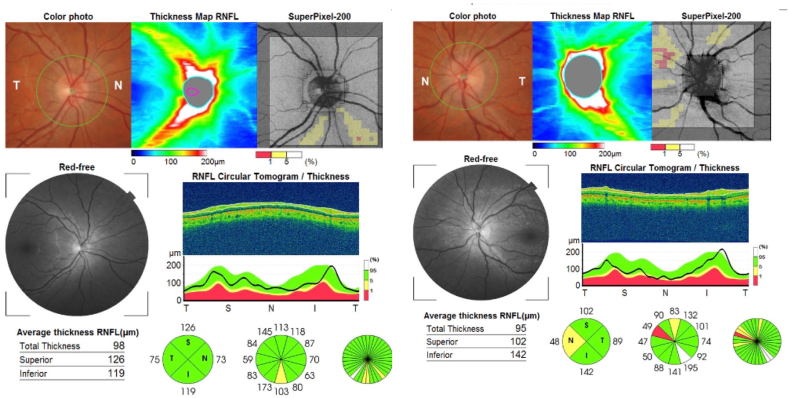
Spectral-domain optical coherence tomography (Cirrus 5000) ten weeks after initial assessment shows resolution of the retinal nerve fiber layer thickening in the left eye and is normal thickness in the right eye.

A 32-year old man who was examined around the same time as his brother (Case 2) presented with a history of transient visual obscurations and headaches which started 3 months before presentation. His past medical history was non-contributory and he not using any medications. His body weight was 82 kg and his height was 175 cm (BMI 24.7). His visual acuity was 20/20 OU with normal color vision testing. Fundus examination showed papilledema in the left eye only (Fig. 6) and his visual field testing showed an enlarged blind spot in the left eye. MRI/MRV of the brain was normal and lumbar puncture done in lateral decubitus position showed an opening pressure of 29 cmH20 with normal CSF analysis. He was started on oral acetazolamide 1 g/day and during follow up he has lost approximately 12 kg of body weight. Ten weeks later he was followed up and his papilledema had resolved in the left eye (Fig. 7).

## 2 Discussion

We report a case series of familial IIH in three non-obese patients (one father and three offsprings) with variable phenotypic presentation as two of the reported cases (Case 3 and 4) had unilateral or asymmetric papilledema. The positive family history in the father suggests an autosomal dominant pattern of inheritance. Familial IIH has been previously reported with most of the reports occurring in obese family members.^3^, ^4^, ^5^, ^6^ The first case of familial IIH was reported in 1969 by Buchheit et al. but since old reports of IIH predated the era of modern neuro-imaging and relied on pneumoencephalograms to rule out a structural lesion, some of these reports do not comply with the modern revised IIH criteria.^7^ Corbett et al. have reported 27 family members with IIH entirely obese except for one case in his series. The most common pattern of inheritance was mother-to-daughter, which suggested a possible autosomal dominant inheritance pattern.^3^ There was one case of obese heterozygous twin sisters in his series but in one of them the CSF opening pressure was not measured because of a “dry tap”.^3^ The author hypothesized the concept of a “genetic clock” since many of these familial cases reached a specific age before the diseases manifested.^3^ The exact pathophysiologic mechanism underlying IIH is still poorly understood and there may not a single unifying mechanism, but the most widely accepted mechanisms is decreased CSF absorption or increased CSF production.^8^ Based on the reports of familial cases of IIH, some authors have hypothesized that genetic variation (polymorphism) in the AQP1 gene may lead to its up-regulation in the choroid plexus and associated increased CSF production leading to high ICP.^9^ Larger studies are needed to explore the role of genetic variation in AQP-1 and it possible association with familial IIH.^10^^,^^11^ Our cases were non-obese and they had variable clinical presentations as two patients (case 2 and 3) had asymmetric unilateral papilledema. Since unilateral or asymmetric papilledema can occur rarely even in non-familial IIH, it might reflect congenital or possibly inherited variation in the elasticity of the optic nerve sheath and therefore asymmetric threshold intracranial pressure to interfere with the axoplasmic flow and cause papilledema. Finally, the BMI in our cases was in the non-obese range (BMI 20.6–24.7), which would favor the possibility of genetic predisposition rather than obesity. This is also supported by previous reports of non-obese familial IIH.^4^, ^5^, ^6^^,^^12^ Karaman et al. have reported six nonobese family members with IIH based on papilledema, however, three of them were diagnosed “presumptively” as they declined lumbar puncture.^5^ Beri et al. have reported a mother and two daughters with IIH with no obesity; the first daughter with typical IIH presentation with papilledema and high CSF opening pressure while the second daughter had documented high CSF opening pressure but no papilledema.^6^ In summary, the absence of obesity in some cases of familiar IIH suggests an underlying genetic cause and a lower threshold for screening symptomatic family members and relatives of IIH patients is warranted in some cases.

## Patient consent

Consent to publish this case report has been obtained from the patients and this report does not contain any personal identifying information.

## Funding

No funding was given.

## Authorship

All authors attest that they meet the current ICMJE criteria for Authorship.

## Declaration of competing interest

None of the authors has any conflicts of interest.
